# Prognostic models for early and late tumor progression prediction in nasopharyngeal carcinoma: An analysis of 8292 endemic cases

**DOI:** 10.1002/cam4.5361

**Published:** 2022-10-27

**Authors:** Lu‐Lu Zhang, Wei‐hong Zheng, Wei‐jie Zhu, Qi‐Ling Deng, Jun‐Ling Peng, Yi‐Yang Li, Ying Sun, Li Lin

**Affiliations:** ^1^ Department of Molecular Diagnostics Sun Yat‐sen University Cancer Center, State Key Laboratory of Oncology in South China, Guangdong Key Laboratory of Nasopharyngeal Carcinoma Diagnosis and Therapy, Collaborative Innovation Center for Cancer Medicine Guangzhou People's Republic of China; ^2^ Department of Radiation Oncology, Sun Yat‐sen University Cancer Center, State Key Laboratory of Oncology in South China, Collaborative Innovation Center for Cancer Medicine Guangdong Key Laboratory of Nasopharyngeal Carcinoma Diagnosis and Therapy Guangzhou People's Republic of China; ^3^ Department of Oncology First affiliated Hospital of Guangdong Pharmaceutical University Guangzhou People's Republic of China

**Keywords:** early tumor progression, late tumor progression, nasopharyngeal carcinoma, nomogram, prognosis

## Abstract

**Objectives:**

The time for posttreatment tumor progression differs between nasopharyngeal carcinoma (NPC) patients. Herein, we established effective nomograms for predicting early tumor progression (ETP) and late tumor progression (LTP) in NPC patients.

**Methods:**

We retrospectively enrolled 8292 NPC patients (training cohort: *n* = 6219; validation cohort: *n* = 2073). The ELP and LTP were defined as the time to tumor progression ≤24 and >24 months after treatment, respectively.

**Results:**

The ETP and LTP accounted for 52.6 and 47.4% of the total patient cohort, respectively. Patients who developed ETP had markedly worse overall survival (OS) versus patients who suffered from LTP (5‐year OS: 26.2% vs. 59.7%, *p* < 0.001). Further, we identified 10/6 predictive factors significantly associated with ETP/LTP via logistic regression analyses. These indicators were used separately to construct two predictive nomograms for ETP and LTP. In the training group, the ETP nomogram [Harrell Concordance Index (C‐index) value: 0.711 vs. 0.618; *p* < 0.001] and LTP nomogram (C‐index value: 0.701 vs. 0.612; *p* < 0.001) were significantly superior for risk stratification than the TNM staging. These results were supported in the validation group with a C‐index value of 0.753 and 0.738 for the ETP and LTP nomograms, respectively. High‐risk patients defined by ETP/LTP nomograms had shorter progression‐free survival than low‐risk patients (all *p* < 0.001).

**Conclusion:**

The established nomograms can help in ELP or LTP risk stratification for NPC patients. Our current results might also provide insights into individualized treatment decisions and designing surveillance strategies for NPC patients.

## Graphical abstract

We successfully constructed and validated two nomograms to facilitate early tumor progression (ETP) and late tumor progression (LEP) risk stratification for NPC patients. The ETP and LTP nomograms demonstrated that the high‐isk group had a shorter progression‐free survival (PFS) than the low‐risk group. Moreover, this study facilitated the prediction of ETP or LTP risk, which might also provide valuable insights into treatment decisions and designing surveillance strategies for NPC after definitive treatment.

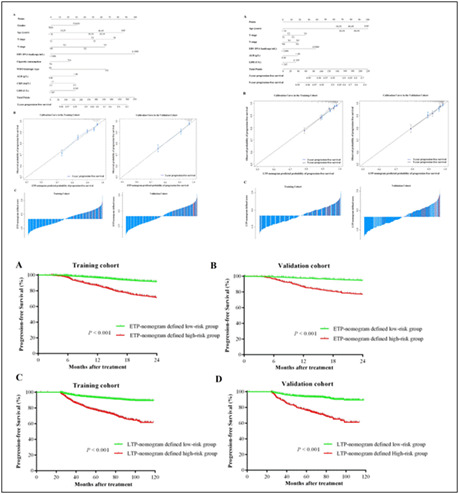

## 1 INTRODUCTION

Nasopharyngeal carcinoma (NPC) has endemic characteristics. For example, more than 70% of newly diagnosed NPC cases are concentrated in Southeast and East Asia.^1^, ^2^ Radical radiotherapy is the primary treatment method for NPC since the tumor is in an anatomically complex region and is radiosensitive.^3^ During the past few decades, the broader clinical utility of precise diagnostic imaging technology and superior radiotherapy combined with chemotherapy have improved the prognosis of NPC patients.^4^, ^5^ However, the long‐term prognosis of NPC remains to be improved, with up to 33% of patients suffering from distant metastasis/locoregional recurrence 10 years after definitive treatment.^6^

After treatment completion, up to 54.8% of tumor progression is observed within 24 months for NPC. Additionally, NPC failure hazard rate varies over time, with a sharp peak at 24 months followed by a decline.^7^ In this context, tumor progression within 24 months is defined as early tumor progression (ETP) and, after this period, as late tumor progression (LTP). A shorter time for tumor progression is usually associated with poorer survival rates in different solid human cancers.^8^, ^9^, ^10^, ^11^, ^12^, ^13^ The biological mechanisms underlying ETP and LTP can differ and might display various failure patterns and prognostic factors. However, research has rarely focused on ETP and LTP for NPC patients.

Given the poor disease control measures and fatal late complications, salvage treatment of metastatic/recurrent NPC is challenging.^14^ Therefore, pretreatment prediction of ETP and LTP is critical to promote early treatment interventions and develop a personalized follow‐up appointment schedule. The TNM staging system defines NPC based on anatomical structure involvement and is the basis for predicting prognosis and therapeutic decisions.^15^ Since the currently used anatomy‐based staging system does not consider biological variability within the tumor, there is inconsistency in prognostic results among patients receiving the same treatment and with the same clinical TNM stage.^16^ Previous studies have reported that other factors, such as gender, age, Epstein–Barr virus deoxyribonucleic acid (EBV DNA), serum lactate dehydrogenase (LDH), albumin (ALB), hemoglobin (HGB), C‐reactive protein (CRP),^17^, ^18^, ^19^, ^20^, ^21^, ^22^ family history of cancer,^23^, ^24^ histological type,^25^, ^26^ alcohol consumption,^27^ and cigarette consumption^28^ contribute to the prognosis of NPC patients. Hence, developing more accurate tools that integrate clinical TNM stage and clinical andmolecular indicators are essential for individualized prognostic prediction and treatment decisions.

Herein, we investigated the prognostic indicators of ETP and LTP after definitive treatment for NPC in an endemic area. Moreover, we constructed more accurate prognostic prediction models by combining TNM staging with clinical and molecular variables. The models were superior for risk stratification than TNM staging and might facilitate pretreatment prediction of ETP and LTP.

## 2 MATERIALS AND METHODS

### 2.1 Patient selection and data extraction

A big data intelligence platform able to facilitate real‐world NPC research was previously constructed at the Sun Yat‐sen University Cancer Center (SYSUCC).^29^ This dynamically updated platform enables real‐time automatic linking, extracting, structuring, and updating routine healthcare data obtained from 13 clinical business systems, including hospital information, electronic medical records, laboratory information, ultrasound, electrocardiogram, pathology, electrocardiogram, endoscopy, MOSAIQ radiotherapy management, anesthesia information management, physical examination information, follow‐up, tumor bio‐bank, and radiation data.

Using the big data intelligence platform, we retrospectively extracted data from 10,126 patients who were newly diagnosed and received radical treatment at the SYSUCC from 2011 to 2015. Eligibility criteria included (1) pathologically confirmed, nondisseminated NPC; (2) complete baseline evaluation and pretreatment clinicopathological data; (3) intact follow‐up data; and (4) treated with definitive intensity‐modulated radiation therapy ± chemotherapy. Finally, 8292 NPC patients were enrolled in this research. We randomly assigned eligible patients to training (*n* = 6219) and validation (*n* = 2073) groups according to a 2:1 ratio using computer‐generated random numbers.

### 2.2 Diagnosis and treatment

Before treatment, the baseline evaluations included an intact physical examination, complete medical history evaluation, plasma EBV DNA load, biochemistry and hematology analysis, histopathological diagnosis, fiberoptic nasopharyngoscopy, magnetic resonance imaging (MRI) of head and neck to the collarbone for primary tumor staging, abdominal ultrasonography, skeletal scintigraphy, chest radiography, and enhanced computed tomography (CT). We restaged patients using the 8th edition of the clinical TNM stage. Treatment regimens are summarized in the Data S1.

### 2.3 Follow‐up and definition of early and late tumor progression

After treatment, patients needed to attend regular follow‐up examinations once per 3 months for the first 36 months, once per 6 months for the next 24 months, then once per 12 months. Follow‐up visits were conducted to detect possible ETP and LTP. They consisted of detailed medical history evaluation, biochemistry and hematology profiles, complete physical examination, plasma EBV DNA load, electronic nasopharyngoscopy, enhanced computed tomography or chest radiography, abdominal ultrasound or CT, skeletal scintigraphy, and head and neck MRI. Additional PET/CT examinations were recommended when necessary.

The endpoint of this study was progression‐free survival (PFS; time from treatment initiation to first tumor progression or death due to any cause or last follow‐up). Treatment progression was defined by detecting a newly confirmed distant metastasis or/and locoregional recurrence. Suspected lesions of distant metastasis and locoregional recurrence were confirmed by imaging examination and/or cytological biopsies. The follow‐up time was calculated from treatment initiation to the last follow‐up visit or death. In the current research, locoregional recurrence/metastasis at ≤24 months and >24 months after definitive treatment for NPC were defined as ETP and LTP, respectively.

### 2.4 Statistical analysis

Statistical analyses were conducted using R (R Core Team, Vienna, Austria, v. 3.4.4) or SPSS (IBM Corp, v.23.0). Thirteen baseline covariates, which have been reported to contribute to the prognosis of NPC patients, were included in the survival analysis. For categorical variables, differences were compared using Fisher's exact or *χ* ^2^ tests. Using clinically recognized cutoff points, descriptive statistics of continuous variables were converted to categorical variables.^17^, ^20^, ^22^, ^30^, ^31^ Univariate and multivariate Cox proportional hazard models were applied to estimate the effects of predictors on ETP and LTP. A forest plot was created to summarize pooled adjusted hazard ratios (HRs) and the 95% confidence intervals (CIs) of significant variables in the univariate analyses. Finally, nomograms for predicting ETP/LTP were generated using Cox regression coefficients of independent risk factors in the multivariate analysis. The score for single risk factors was calculated by mapping points on a scale axis. The sum score of an individual patient was determined as the sum of scores for all independent risk factors. Finally, we calculated the ETP or LTP likelihood for each patient. We calculated survival rates using Kaplan–Meier curves, and differences in characteristics were examined by the log‐rank test. The performance and predictive reliabilities of the nomograms were assessed using Harrell's concordance index (C‐index). Calibration plots were drawn to reflect the accuracy between predicted PFS and actual observed survival. Receiver‐operating characteristic (ROC) curves were generated to assess the specificity and sensitivity of the nomograms. The area under the ROC curve (AUC) or C‐index ranged from 0.5 to 1.0, with 0.5 indicating the random chance of the model correctly predicting outcomes and 1.0 indicating perfect predictive performance. The survival difference between nomogram‐defined risk groups was evaluated using the log‐rank test. A *p* < 0.05 indicated statistical significance.

### 2.5 Ethical statement

The Institutional Review Board and Ethics Committee of our institution approved the current retrospective analysis using anonymous data. They also waived the requirement to obtain written informed consent from patients, given the study's retrospective nature (approval number: B2020‐267).

## 3 RESULTS

### 3.1 General situation

Among the 8292 patients enrolled, 6219 and 2073 patients were assigned to the training and validation sets. The baseline demographics and clinical characteristics of the two sets are listed in Table S1. The median follow‐up time was 67.7 months (2.0–123.0 months), 67.8 months (2.6–123.0 months), and 67.4 months (2.0–118.1 months) for the total, training, and validation sets, respectively. After definitive treatment, 23.6% (1953/8292) patients suffered tumor progression in the whole cohort, including 52.6% (1027/1953) with the first tumor progression within 24 months and 47.4% (926/1953) with the first tumor progression after ≥24 months. The comparison of clinicopathological factors between patients who suffered ETP and LTP is presented in Table 1.

**TABLE 1:** Comparison of baseline clinical and treatment characteristics among nasopharyngeal carcinoma patients who experienced early/late tumor progression

Characteristic	Training cohort (*N* = 6219)				Validation cohort (*N* = 2073)			
	Early tumor progression group (*N* = 774, %)	Late tumor progression group (*N* = 695, %)	Disease‐free group (*N* = 4750, %)	*p* value^a^	Early tumor progression group (*N* = 253, %)	Late tumor progression group (*N* = 231, %)	Disease‐free group (*N* = 1589, %)	*p* value^a^
Age (years)				< 0.001				< 0.001
<18	5 (2.2)	1 (10.9)	40 (87.0)		1 (6.7)	0 (0.0)	14 (93.3)	
18–29	42 (9.0)	40 (8.6)	383 (82.4)		4 (2.8)	8 (5.6)	132 (91.7)	
30–39	171 (11.9)	115 (8.0)	1149 (80.1)		49 (9.7)	48 (9.5)	406 (80.7)	
40–49	228 (10.7)	265 (12.4)	1636 (76.8)		93 (12.6)	73 (9.9)	571 (77.5)	
50–59	166 (11.5)	185 (12.9)	1088 (75.6)		61 (13.3)	59 (12.9)	337 (73.7)	
≥60	125 (17.7)	126 (17.9)	454 (64.4)		45 (20.7)	43 (19.8)	129 (59.4)	
Gender				0.001				< 0.001
Male	611 (13.3)	496 (10.8)	3486 (75.9)		215 (14.0)	197 (12.8)	1128 (73.2)	
Female	163 (10.0)	199 (12.2)	1264 (77.7)		38 (7.1)	34 (6.4)	461 (86.5)	
WHO histologic type				< 0.001				0.001
Type I–II	36 (23.5)	19 (12.4)	98 (64.1)		14 (29.2)	7 (14.6)	27 (56.3)	
Type III	738 (12.2)	676 (11.1)	4652 (76.7)		239 (11.8)	224 (11.1)	1562 (77.1)	
T stage^b^				< 0.001				< 0.001
T1	59 (5.5)	73 (6.8)	948 (87.8)		16 (4.7)	21 (6.1)	306 (89.2)	
T2	117 (11.6)	93 (9.2)	796 (79.1)		38 (11.4)	37 (11.1)	257 (77.4)	
T3	361 (12.6)	320 (11.2)	2188 (76.3)		111 (11.5)	93 (9.6)	763 (78.9)	
T4	237 (18.8)	209 (16.5)	818 (64.7)		88 (20.4)	80 (18.6)	263 (61.0)	
N stage^b^				< 0.001				< 0.001
N0	60 (6.1)	65 (6.6)	857 (87.3)		11 (3.0)	22 (6.0)	335 (91.0)	
N1	342 (10.8)	344 (10.9)	2478 (78.3)		105 (10.3)	105 (10.3)	813 (79.5)	
N2	200 (15.3)	179 (13.7)	929 (71.0)		82 (18.6)	61 (13.8)	298 (67.6)	
N3	172 (22.5)	107 (14.0)	486 (63.5)		55 (22.8)	43 (17.8)	143 (59.3)	
TNM stage^b^				< 0.001				< 0.001
I	6 (1.6)	13 (3.6)	345 (94.8)		1 (0.7)	7 (5.0)	133 (94.3)	
II	87 (7.7)	85 (7.6)	952(84.7)		27 (7.4)	33 (9.0)	306 (83.6)	
III	314 (11.0)	305 (10.7)	2238 (78.3)		100 (10.5)	85 (8.9)	770 (80.6)	
IV	367 (19.6)	292 (15.6)	1215 (64.8)		125 (20.5)	106 (17.3)	380 (62.2)	
Cigarette consumption				< 0.001				< 0.001
No	443 (11.0)	420 (10.4)	3163 (78.6)		141 (10.4)	140 (10.3)	1080 (79.4)	
Yes	331 (15.1)	275 (12.5)	1587 (72.4)		112 (15.7)	91 (12.8)	509 (71.5)	
Alcohol consumption				0.062				0.771
No	648 (12.1)	585 (11.0)	4103 (76.9)		215 (12.0)	197 (11.0)	1373 (76.9)	
Yes	126 (14.3)	110 (12.5)	647 (73.3)		38 (13.2)	34 (11.8)	216 (75.0)	
Family of cancer history				0.644				0.504
No	577 (12.6)	521 (11.3)	3493 (76.1)		185 (12.5)	170 (11.5)	1122 (76.0)	
Yes	197 (12.1)	174(10.7)	1257 (77.2)		68 (11.4)	61 (10.2)	467 (78.4)	
EBV DNA load, copy/mL				< 0.001				< 0.001
<2000	173 (5.6)	185 (6.0)	2720 (88.4)		50 (4.9)	52 (5.1)	913 (90.0)	
≥ 2000	601 (19.1)	510 (16.2)	2030 (64.6)		203 (19.2)	179 (16.9)	676 (63.9)	
HGB (g/L)				0.322				0.594
<120	60 (14.3)	52 (12.4)	309 (73.4)		21 (14.4)	18 (12.3)	107 (73.3)	
≥120	714 (12.3)	643 (11.1)	4441 (76.6)		232 (12.0)	213 (11.1)	1482 (76.9)	
LDH (U/L)				< 0.001				< 0.001
<245	671 (11.7)	601 (10.5)	4444 (77.7)		210 (11.0)	192 (10.1)	1500 (78.9)	
≥245	103 (20.5)	94 (18.7)	306 (60.8)		43 (25.1)	39 (22.8)	89 (52.0)	
ALB (g/L)				< 0.001				< 0.001
<40	120 (19.8)	108 (17.8)	378 (62.4)		45 (24.5)	35 (19.0)	104 (56.5)	
≥40	654 (11.7)	587 (10.5)	4372 (77.9)		208 (11.0)	196 (10.4)	1458 (78.6)	
CRP (mg/L)				< 0.001				< 0.001
<1.0	187 (9.4)	195 (9.8)	1610 (80.8)		65 (9.1)	73 (10.3)	574 (80.6)	
1.0–3.0	251 (10.9)	269 (11.7)	1783 (77.4)		76 (9.6)	93 (11.7)	624 (78.7)	
≥3.0	336 (17.5)	231 (12.0)	1357 (70.5)		112 (19.7)	65 (11.4)	391 (68.8)	

Abbreviations: ALB, albumin; CRP, C‐reactive protein; EBV DNA, circulating cell‐free Epstein–Barr virus deoxyribonucleic acid; HGB, hemoglobin; LDH, lactate dehydrogenase; WHO, World Health Organization.

^a^ Statistical comparisons between the training cohort and validation cohort were computed using the Chi‐square test or Fisher's exact test. A *p* value of 0.05 indicates a significant difference.

^b^ According to the 8th edition of the AJCC/UICC staging system.

### 3.2 ETP and LTP are correlated with differential overall survival in patients with NPC

**FIGURE 1:**
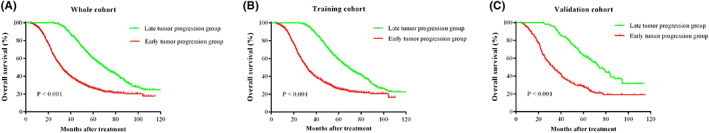
Kaplan–Meier plots of overall survival outcomes for nasopharyngeal carcinoma patients who experienced early/late tumor progression. (A) Whole cohort, *n* = 8292; (B) training cohort, *n* = 6219; (C) validation cohort, *n* = 2073.

The estimated 1‐, 3‐, and 5‐year OS rates were 99.2, 92.1, and 86.1% in the training set; 99.0, 92.5, and 87.1% in the validation set; 99.2, 92.2, and 86.4% in the entire set, respectively. For the ETP and LTP groups, the median OS time were 31.9 (95% CI: 30.1–33.7) and 69.9 (95% CI: 66.3–73.5) months in the entire set; 31.7 (95% CI: 29.7–33.7) and 68.6 (95% CI: 64.4–72.8) months in the training set; 32.0 (95% CI: 28.3–37.8) and 74.2 (95% CI: 66.8–81.6) months in the validation set. The 5‐year OS rates of the ETP and LTP groups were 25.7 and 58.5% for the training set (*p* < 0.001); 28.0 and 63.5% for the validation set (*p* < 0.001); 26.2 and 59.7% for the entire set (*p* < 0.001). The Kaplan–Meier analyses revealed that the OS for patients who suffered ETP was worse than for patients who suffered LTP (Figure 1).

### 3.3 Pretreatment risk factors associated with ETP

Next, we determined the prognostic significance of the 13 baseline covariates (T stage, N stage, gender, age, World Health Organization [WHO] histologic type, CRP, ALB, HGB, LDH, EBV DNA load, family history of cancer, histological type, alcohol consumption, and cigarette consumption) using the Cox‐proportional hazards model. The univariate analyses determined 10 potential predictors of ETP in the training set: T stage, N stage, gender, age, WHO histologic type, CRP, ALB, LDH, EBV DNA load, and cigarette consumption (Figure S1). In the multivariate analysis, these 10 covariates were also independent risk factors for NPC patients with ETP (Table S2).

### 3.4 Nomogram for ETP prediction

**FIGURE 2:**
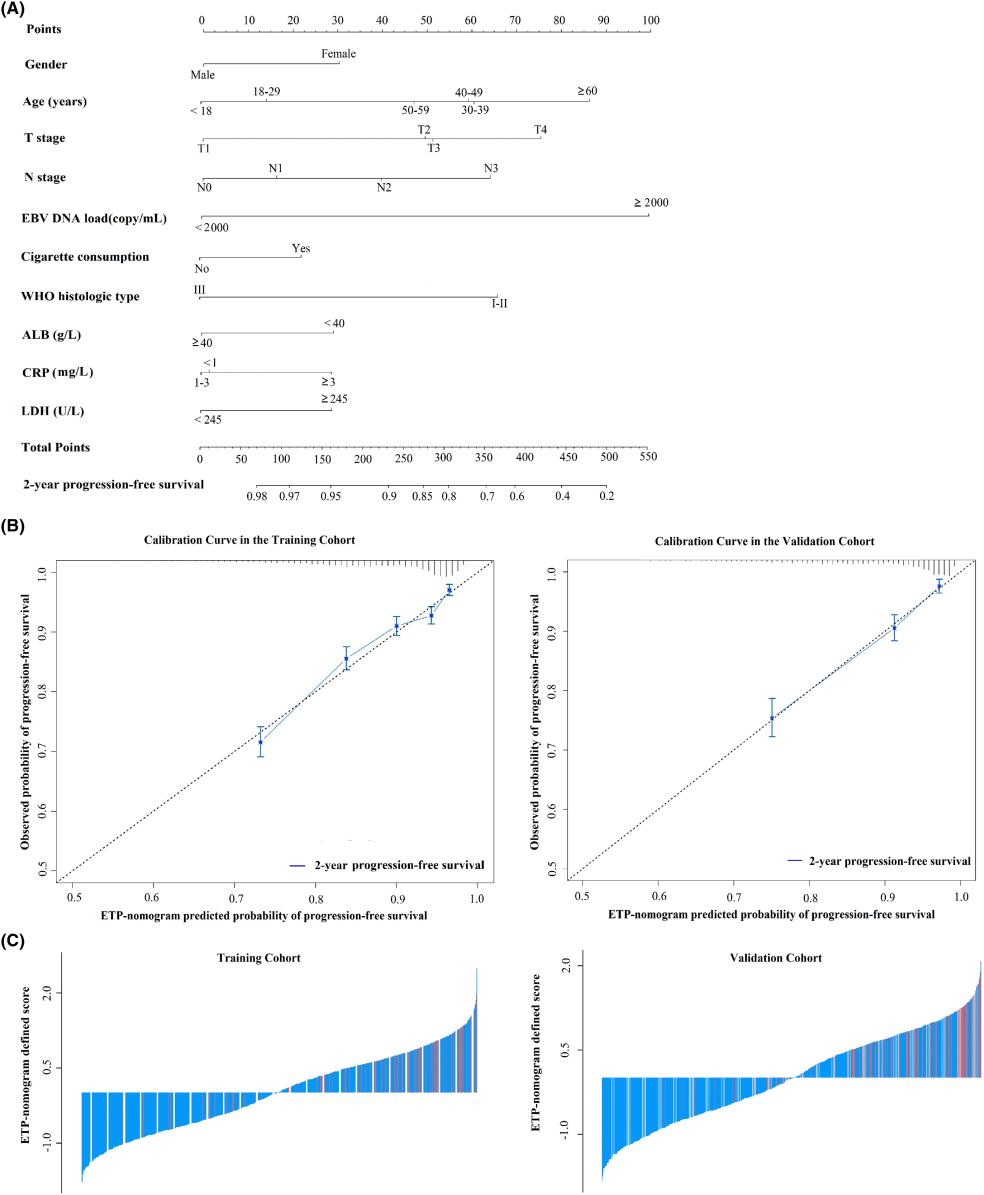
Nomogram to predict early tumor progression, calibration curves, and nomogram‐defined score. (A) Nomogram to predict ETP in NPC patients. (B) Calibration curves of the nomogram to predict ETP in the training (*n* = 6219) and validation cohort (*n* = 2073). Nomogram‐predicted probability of 2‐year PFS is plotted on the *x*‐axis; the actual observed probability is plotted on the *y*‐axis. (C) Nomogram‐defined score for each NPC patient in the training (*n* = 6219) and validation (*n* = 2073) cohorts. The red bars represent the scores for those experiencing ETP, whereas the blue bars represent the scores for patients who did not experience ETP. (B) ALB, albumin; CRP, C‐reactive protein; EBV DNA, circulating cell‐free Epstein–Barr virus deoxyribonucleic acid; ETP, early tumor progression; LDH, lactate dehydrogenase; NPC, nasopharyngeal carcinoma; PFS, progression‐free survival; WHO, World Health Organization.

The independent prognostic factors identified in the multivariate analysis for the training cohort were incorporated into the ETP nomogram to visually quantify the ETP probability (Figure 2A). For the training and validation sets, the calibration curves for early PFS prediction showed a good agreement between nomogram prediction and the actual observed early PFS probabilities (Figure 2B). The sum of scores of each single risk factor determined using the nomogram was used to calculate the total score for an individual case. The detailed risk score distribution is shown in Figure 2C.

For the training (C‐index: 0.711; 95% CI: 0.694–0.729) and validation (C‐index, 0.753; 95% CI: 0.725–0.780) sets, the ETP nomogram exhibited a high accuracy for ETP prediction. Similarly, the AUC of the ETP nomogram was 0.711 in the training set and 0.753 in the validation set for ETP prediction. These results indicated the good discrimination ability for prognosis prediction using the ETP nomogram.

Further, we compared our models with the eighth TNM stage and 10 single risk factors. For the training and validation sets, the results showed that the C‐index of the ETP nomogram for ETP prediction was 0.711 (95% CI: 0.694–0.729) and 0.753 (95% CI: 0.725–0.780), statistically higher than the C‐index of the eighth clinical TNM stage and single risk factors of T stage, N stage, gender, age, WHO histologic type, CRP, ALB, LDH, EBV DNA load, and cigarette consumption (*p* < 0.05; Table 2).

**TABLE 2:** Summary of the C‐index of prognostic models and single risk factors for early/late tumor progression prediction in nasopharyngeal carcinoma

Risk factors	C‐index (95% CI)					
	Early tumor progression prediction in the training cohort	Early tumor progression prediction in the validation cohort	*p* value^†^	Late tumor progression prediction in the training cohort	Late tumor progression prediction in the validation cohort	*p* value^†^
Prognostic models						
ETP nomogram/LTP nomogram	0.711 (0.694,0.729)	0.753 (0.725,0.780)	Reference	0.701 (0.682,0.721)	0.738 (0.709,0.767)	Reference
Eighth TNM stage	0.618 (0.600,0.635)	0.635 (0.605,0.665)	<0.001	0.612 (0.592,0.631)	0.626 (0.591,0.661)	<0.001
Single risk factors						
Gender	0.528 (0.513,0.542)	0.558 (0.535,0.580)	<0.001	/	/	/
Age	0.532 (0.512,0.552)	0.584 (0.551,0.617)	<0.001	0.571 (0.550,0.593)	0.590 (0.553,0.627)	<0.001
T stage	0.589 (0.571,0.608)	0.606 (0.575,0.638)	<0.001	0.598 (0.578,0.618)	0.617 (0.581,0.652)	<0.001
N stage	0.604 (0.586,0.623)	0.646 (0.616,0.676)	<0.001	0.585 (0.565,0.605)	0.603 (0.568,0.638)	<0.001
EBV DNA load	0.648 (0.633,0.662)	0.655 (0.630,0.681)	<0.001	0.644 (0.627,0.661)	0.655 (0.626,0.684)	<0.001
Cigarette consumption	0.541 (0.524,0.559)	0.554 (0.524,0.585)	<0.001	/	/	/
WHO histologic type	0.512 (0.505,0.519)	0.516 (0.503,0.530)	<0.001	/	/	/
ALB	0.532 (0.519,0.544)	0.548 (0.525,0.571)	<0.001	0.531 (0.518,0.544)	0.547 (0.522,0.571)	<0.001
CRP	0.575 (0.555,0.594)	0.594 (0.559,0.628)	<0.001	/	/	/
LDH	0.530 (0.518,0.542)	0.549 (0.526,0.572)	<0.001	0.529 (0.516,0.541)	0.551 (0.527,0.574)	<0.001

Abbreviations: ALB, albumin; CRP, C‐reactive protein; CI: confidence intervals; C‐index, Harrell's concordance indices; cf EBV DNA, circulating cell‐free Epstein–Barr virus deoxyribonucleic acid; ETP: early tumor progression; LDH, lactate dehydrogenase; LTP: late tumor progression.

^†^ A *p* value of 0.05 indicates a significant difference.

**FIGURE 3:**
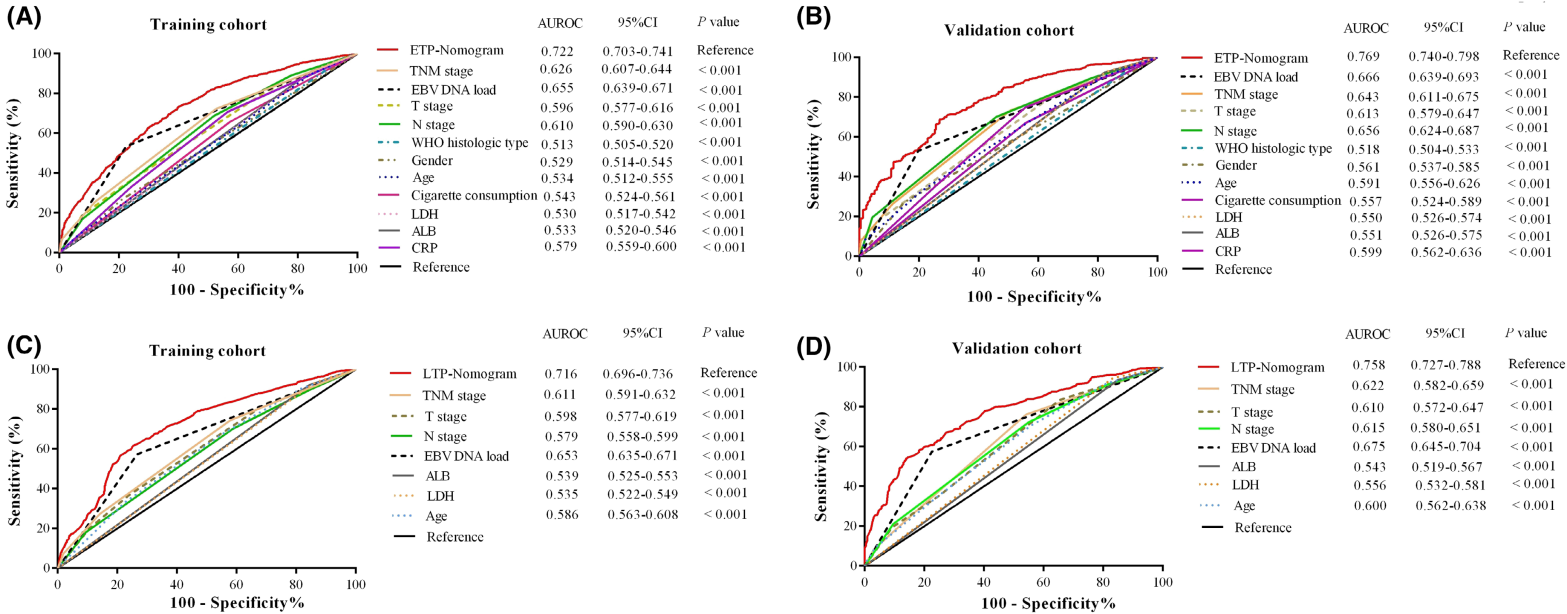
Receiver‐operating characteristic curves to compare the predictive power of ETP nomogram (A, B)/LTP nomogram (C, D), TNM stage, and single risk factors for predicting early tumor progression in nasopharyngeal carcinoma. ALB, albumin; AUC, area under the receiver operator characteristic curve; CRP, C‐reactive protein; cf EBV DNA, circulating cell‐free Epstein–Barr virus deoxyribonucleic acid; ETP, early tumor progression; HGB, hemoglobin; LDH, lactate dehydrogenase; LTP, late tumor progression; WHO, World Health Organization.

The AUCs of the ETP nomogram (training set: 0.722, 95% CI: 0.703–0.741; validation set: 0.769, 95% CI: 0.740–0.798) were superior than the eighth clinical TNM stage and single risk factors for ETP prediction (Figure 3A,B).

### 3.5 Pretreatment risk factors associated with LTP

After excluding 1027 cases who suffered ETP, we included 7265 cases in the univariate and multivariate analyses to determine pretreatment risk factors associated with LTP. In the univariate analysis, eight covariates (T stage, N stage, age, CRP, ALB, LDH, plasma EBV DNA load, and cigarette consumption) were identified as significant prognostic factors for LTP (*p* < 0.05). With the multivariate analysis adjustment, six covariates (T stage, N stage, age, ALB, LDH, and plasma EBV DNA load) significantly affected LTP (*p* < 0.05). Detailed results are shown in Figure S2 and Table S2.

### 3.6 Nomogram for LTP

**FIGURE 4:**
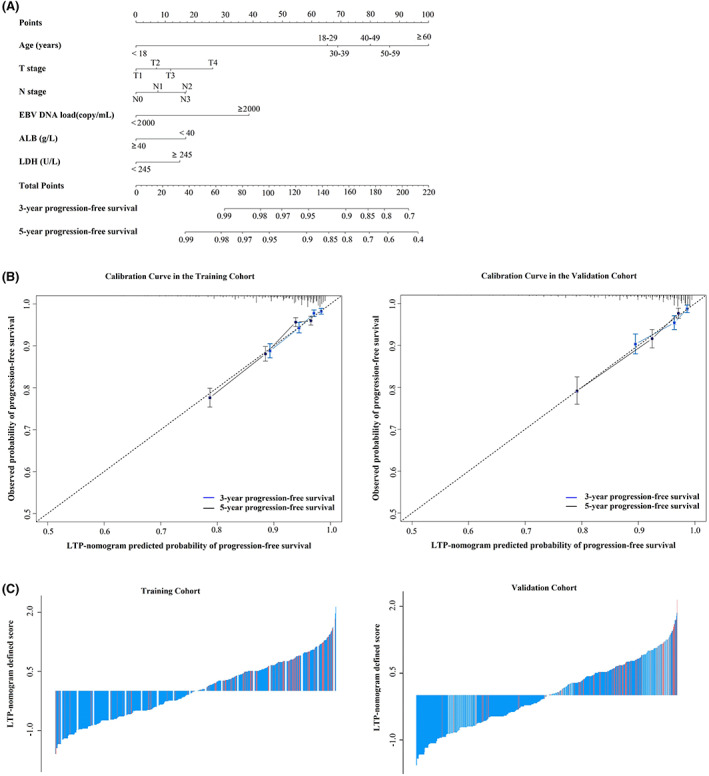
Nomogram to predict late tumor progression, calibration curves, and nomogram‐defined score. (A) Nomogram to predict LTP in NPC patients. (B) Calibration curves of the nomogram to predict LTP after 2 years in the training (*n* = 5445) and validation (*n* = 1820) cohorts. Nomogram‐predicted probability of 3‐year and 5‐year PFS is plotted on the *x*‐axis; the actual observed probability is plotted on the *y*‐axis. (C) Nomogram‐defined score for each NPC patient in the training (*n* = 5445) and validation (*n* = 1820) cohorts. The red bars represent scores of patients who experienced LTP, whereas the blue bars represent the scores of patients who did not experience LTP. ALB, albumin; EBV DNA, circulating cell‐free Epstein–Barr virus deoxyribonucleic acid; LDH, lactate dehydrogenase; LTP, late tumor progression; NPC, nasopharyngeal carcinoma; PFS, progression‐free survival.

The LTP nomogram was constructed to predict individual LTP probability according to the multivariate data analysis (Figure 4A). An appropriate degree of consistency was detected between the actual and predicted probability of LTP, and the calibration curve was close to the reference line **(**Figure 4B). The total scores for individual patients are shown in Figure 4C. For LTP prediction, the C‐indices of the LTP nomogram (training set: 0.701, 95% CI: 0.682–0.721; validation set: 0.738, 95% CI: 0.709–0.767) were superior compared with the eighth clinical TNM stage, T stage, N stage, age, ALB, LDH, and plasma EBV DNA load (*p* < 0.05; Table 2).

Similarly, the LTP nomogram presented significantly higher AUC values (0.716, 95% CI: 0.696–0.736; 0.758, 95% CI: 0.727–0.788) compared with single risk factors of T stage, N stage, age, LDH, ALB, EBV DNA load, and the eighth clinical TNM stage for LTP prediction (Figure 3C,D).

### 3.7 Risk stratification for ETP and LTP

**FIGURE 5:**
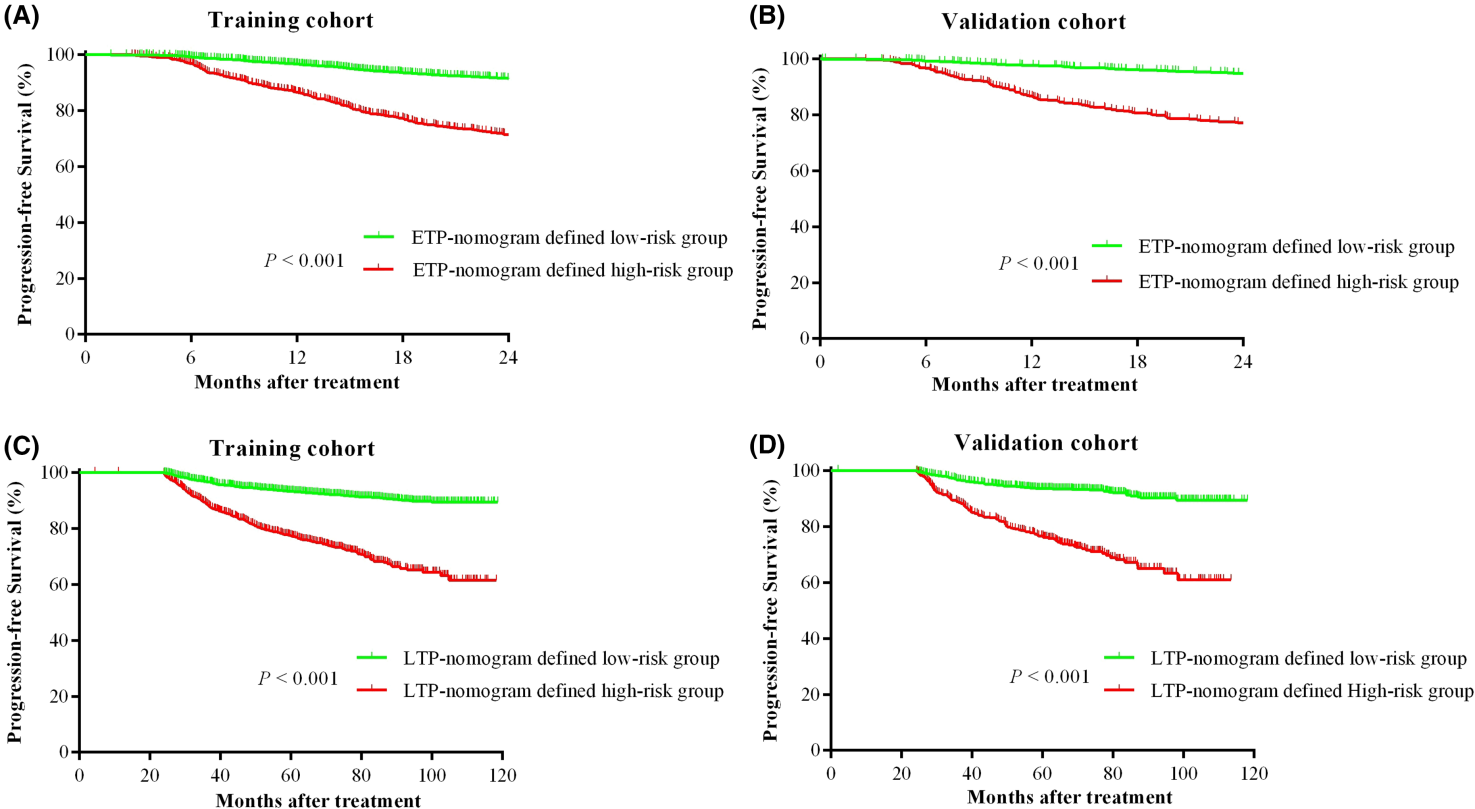
Kaplan–Meier survival curves show that the ETP‐nomogram/LTP‐nomogram‐generated subgroups result in significant differentiation based on PFS. PFS of the ETP‐nomogram‐generated high‐risk and low‐risk groups in the (A) training cohort and (B) validation cohort; PFS of the LTP‐nomogram‐generated high‐risk and low‐risk group in the (C) training cohort and (D) validation cohort. ETP, early tumor progression; LTP, late tumor progression; PFS, progression‐free survival.

According to the scores obtained using the ETP nomogram in the training set, patients were categorized into either low‐ or high‐risk (risk score <1.8 or ≥1.8) groups using an X‐tile (Figure S3). A total of 5011 (80.6%) and 1208 (19.4%) cases in the training set, and 1627 (78.5%) and 446 (21.5%) cases in the validation set were classified into low‐ and high‐risk groups, respectively. The survival analysis demonstrated that the actual PFS within 2 years significantly differed between the ETP nomogram‐defined risk groups (*p* < 0.001; Figure 5A,B).

The best cutoff point of the risk score was 3.4 for the LTP‐prediction nomogram. After excluding 1027 patients with ETP, 7265 cases were classified into low‐ or high‐risk groups (Figure S4). A total of 4078 (74.9%) and 1367 (25.1%) cases in the training set and 1373 (75.4%) and 447 (24.6%) cases in the validation set were assigned to low‐ or high‐risk groups, respectively. The Kaplan–Meier survival analysis demonstrated that the LTP nomogram‐generated subgroups presented significant differences for PFS (Figure 5C,D; *p* < 0.001).

## 4 DISCUSSION

Herein, we used a big data intelligence platform‐generated database to evaluate the incidence, risk factors, and long‐term survival outcomes of ETP or LTP after definitive treatment for NPC. We successfully generated and validated two novel nomograms that can be used to predict ETP or LTP in individual NPC patients. Our current data might also provide valuable insights into treatment decisions and designing rational surveillance strategies for NPC patients.

In this retrospective research, 23.6% of patients in the 8292 patient cohort had ETP and LTP. Most patients suffered tumor progression within 24 months after treatment. Among the 8292 patients, 12.4% (1027/8292; 52.6% of tumor progression) experienced first tumor progression within 24 months after definitive treatment, and 11.2% (926/8292; 47.4% of tumor progression) experienced the first tumor progression after 24 months. Additionally, the disease failure patterns differed among patients with ETP and LTP. Recently, Li et al.^8^ investigated the independent predictors for early recurrence and late‐recurrence NPC patients. However, no previous studies have focused on early and late tumor progression (metastasis/recurrence). We separately investigated risk factors among patients with ETP and LTP, and six (T stage, N stage, age, ALB, LDH, and EBV DNA load) independent risk factors were identified for ETP and LTP. Additionally, the timing of tumor progression might indicate distinct mechanisms or outcomes.^32^, ^33^, ^34^, ^35^, ^36^ Thus, ETP is generally correlated with poor prognosis and aggressive behavior.^8^, ^9^, ^10^, ^11^, ^12^, ^13^ Survival after tumor progression might also reflect the malignant characteristics of cancers. In the present study, the OS of patients with ETP (within 24 months after treatment) was worse than patients who suffered LTP (24 months after definitive treatment for NPC). Understanding the patterns, risk factors, and long‐term prognosis of ETP and LTP might provide insights into the different biological behaviors of these patients.

Thus, differential pretreatment prediction of ETP and LTP might facilitate individualized treatment decisions in a clinical setting and identify rational strategies for monitoring metastasis/recurrence after treatment completion. In our previous research,^13^ we constructed a nomogram model based on clinical variables for solely predicting early metastasis for NPC patients. However, in this study, TNM staging was combined with clinical and molecular variables to establish two separate nomograms (ETP nomogram and LTP nomogram) with enhanced prognostic capability. The ETP and LTP nomograms were superior for risk stratification over TNM staging to facilitate the pretreatment prediction of ETP and LTP risk. Based on the ETP nomogram‐defined score, we assigned individual patients into low‐ETP or high‐ETP risk groups. Similarly, according to the LTP nomogram‐generated score, patients were assigned to low‐LTP‐risk or high‐LTP‐risk groups. The Kaplan–Meier survival curves showed that nomogram‐generated groups significantly differed based on PFS.

Data on risk assessments associated with post‐treatment ETP/LTP might also help direct postoperative surveillance strategies. Our current data indicated that the post‐treatment period should be considered to establish an individualized and more cost‐effective surveillance strategy for NPC patients. Individual patient risk for tumor progression was evaluated using the LTP nomogram for the first 2 years after treatment as well as the ETP nomogram. For example, if a patient was evaluated as high‐risk of ETP using the ETP nomogram, this patient should be on a stringent surveillance program during the follow‐up, allowing timely treatment when tumor progression occurs. If a patient did not suffer tumor progression during the first 2 years after treatment and with a high risk of LTP predicted by the LTP nomogram, this patient would need to be on close and stringent recurrence surveillance 2 years after treatment.

Finally, our study also has some limitations. First, the nomogram model is classic and has been applied in many studies. Thus, the statistical methods applied in this study were not innovative enough. We choose this statistical method because nomograms are a convenient, easy‐to‐use, and reliable prognosis prediction tool for clinicians. Second, given the retrospective and nonrandomized nature, the current results might be affected by selection bias. Notwithstanding this limitation, a large sample size (up to 8292 cases) was used to minimize the bias generated by the retrospective data collection nature. Third, all data were obtained from a single cancer treatment center, limiting the applicability of our findings. Therefore, large‐scale and multicenter studies on other populations are warranted to corroborate these findings further. We are conducting a multicenter prospective study to confirm our findings in the SYSUCC and Wuzhou Red Cross Hospital, and we look forward to sharing the results in the future.

## 5 CONCLUSIONS

ETP and LTP are correlated with differential 5‐year OS of NPC patients (26.2% vs. 59.7%). We successfully constructed and validated two nomograms to facilitate ETP and LTP risk stratification for NPC patients. The ETP and LTP nomograms demonstrated that the high‐risk group had a shorter PFS than the low‐risk group. Moreover, this study facilitated the prediction of ETP or LTP risk, which might also provide valuable insights into treatment decisions and designing surveillance strategies for NPC after definitive treatment.

## AUTHOR CONTRIBUTIONS

**Lu‐lu Zhang:** Conceptualization (equal); funding acquisition (equal); methodology (equal); writing – original draft (equal); writing – review and editing (equal). **Weihong Zheng:** Data curation (equal); formal analysis (equal); investigation (equal); methodology (equal); writing – original draft (equal). **Wei‐Jie Zhu:** Data curation (equal); formal analysis (equal); methodology (equal); writing – original draft (equal). **Qi‐Ling Deng:** Data curation (equal). **Jun‐Ling Peng:** Data curation (equal). **Yi‐Yang Li:** Data curation (equal). **Ying Sun:** Conceptualization (equal); funding acquisition (equal). **Li Lin:** Conceptualization (equal).

## FUNDING INFORMATION

This work was supported by the National Key Research and Development Program of China (grant nos. 2020YFC1316900 and 2020YFC1316901), the National Natural Science Foundation of China (grant nos. 82002220 and 81902762), the China Postdoctoral Science Foundation (grant no. 2019M663305), the National Key R&D Program of China (grant nos. 2020YFC1316900 and No. 2020YFC1316904). The funders had no role in study design, data collection and analysis, decision to publish, or preparation of the manuscript.

## CONFLICT OF INTEREST

The authors have no conflict of interest to declare otherwise.

## ETHICS APPROVAL

The authors are accountable for all aspects of the work in ensuring that questions related to the accuracy or integrity of any part of the work are appropriately investigated and resolved.

## Supporting information


Data S1

## Data Availability

Research data are not shared.
