# Supplementary material for: Prognostic models for early and late tumor progression prediction in nasopharyngeal carcinoma: An analysis of 8292 endemic cases
Source: Cancer Med. 2022 Oct 27;12(5):5384–96. doi: 10.1002/cam4.5361 (PMC10028159; doi:10.1002/cam4.5361)

***Supplementary Materials***

**Treatment regimens**

All eligible 8,292 patients received IMRT as the primary modality of treatment. The implementation of additional chemotherapy were determined based on the institutional treatment guidelines for non-disseminated NPC, which recommended definitive IMRT alone and no chemotherapy for stage I NPC, and concurrent chemoradiotherapy (CCRT) ± neoadjuvant chemotherapy (NACT) or adjuvant chemotherapy (ACT) for stage II to IVa NPC. All patients were treated with fractionated IMRT five days per week. The prescribed total radiation doses were 66–72 Gy for the primary tumor, 64–70 Gy for the cervical lymph nodes, 60–63 Gy for the high-risk region, and 54–56 Gy for the low-risk and neck nodal regions. The NACT or ACT regimens typically included docetaxel/cisplatin/fluorouracil (TPF; 60 mg/m2 d1, 60 mg/m2 d1 and 3000 mg/m2 d1-d5), docetaxel/cisplatin (TP; 75 mg/m2 d1 and 75 mg/m2 d1), cisplatin/fluorouracil (PF; 80 mg/m2 d1 and 4000 mg/m2 d1-d5). NACT/ACT was repeated every 3 weeks for 2-4 cycles. The CCRT regimen, which began on the first day of IMRT, consisted of 30-40 mg/m2 cisplatin administered every week for a maximum of 7 cycles; and 80-100 mg/m2 cisplatin administered at weeks 1, 4, and 7 of the IMRT duration for a maximum of 3 cycles.

**Supplementary Table 1:** Comparison of baseline demographics and clinical characteristics of nasopharyngeal carcinoma patients in the training and validation cohorts.

| **Characteristic** | **Training cohort**  **(N =6,219, %)** | **Validation cohort**  **(N =2,073, %)** | ***P* Value^†^** |
| --- | --- | --- | --- |
| **Age (years)** |  |  | 0.522 |
| **< 18** | 46 (0.7) | 15 (0.7) |  |
| **18–29** | 465 (7.5) | 144 (6.9) |  |
| **30–39** | 1,435 (23.1) | 503 (24.3) |  |
| **40–49** | 2,129 (34.2) | 737 (35.6) |  |
| **50–59** | 1,439 (23.1) | 457 (22.0) |  |
| **≥60** | 705 (11.3) | 217 (10.5) |  |
| **Gender** |  |  | 0.696 |
| **Male** | 4,593 (73.9) | 1,540 (74.3) |  |
| **Female** | 1,626 (26.1) | 533 (25.7) |  |
| **WHO histologic type** |  |  | 0.711 |
| **Type I-II** | 153 (2.5) | 48 (2.3) |  |
| **Type III** | 6,066 (97.5) | 2,025 (97.7) |  |
| **T stage*** |  |  | 0.828 |
| **T1** | 1,080 (17.4) | 343 (16.5) |  |
| **T2** | 1,006 (16.2) | 332 (16.0) |  |
| **T3** | 2,869 (46.1) | 967 (46.6) |  |
| **T4** | 1,264 (20.3) | 431 (20.8) |  |
| **N stage*** |  |  | 0.170 |
| **N0** | 982 (15.8) | 368 (17.8) |  |
| **N1** | 3,164 (50.9) | 1,023 (49.3) |  |
| **N2** | 1,308 (21.0) | 441 (21.3) |  |
| **N3** | 765 (12.3) | 241 (11.6) |  |
| **TNM stage** * |  |  | 0.444 |
| **I** | 364 (5.9) | 141 (6.8) |  |
| **II** | 1,124 (18.1) | 366 (17.7) |  |
| **III** | 2,857 (45.9) | 955 (46.1) |  |
| **IV** | 1,874 (30.1) | 611 (29.5) |  |
| **Cigarette consumption** |  |  | 0.449 |
| **No** | 4,026 (64.7) | 1,361 (65.7) |  |
| **Yes** | 2,193 (35.3) | 712 (34.3) |  |
| **Alcohol consumption** |  |  | 0.729 |
| **No** | 5,336 (85.8) | 1,785 (86.4) |  |
| **Yes** | 883 (14.2) | 288 (13.9) |  |
| **Family of cancer history** |  |  | 0.022 |
| **No** | 4,591 (73.8) | 1,477 (71.2) |  |
| **Yes** | 1,628 (26.2) | 596 (28.8) |  |
| **EBV DNA load, copy/mL** |  |  | 0.676 |
| **< 2000** | 3,078 (49.5) | 1,015 (49.0) |  |
| **≥ 2000** | 3,141 (50.5) | 1,058 (51.0) |  |
| **HGB (g/L)** |  |  |  |
| **<120** | 421 (6.8) | 146 (7.0) | 0.669 |
| **≥120** | 5,798 (93.2) | 1,927 (93.0) |  |
| **LDH (U/L)** |  |  | 0.817 |
| **<245** | 5,716 (91.9) | 1,902 (91.8) |  |
| **≥245** | 503 (8.1) | 171 (8.2) |  |
| **ALB (g/L)** |  |  | 0.244 |
| **<40** | 606 (9.7) | 184 (8.9) |  |
| **≥40** | 5,613 (90.3) | 1,889 (91.1) |  |
|  |  |  |  |
| **CRP (mg/L)** |  |  | 0.008 |
| **<1.0** | 1,992 (32.0) | 712 (34.3) |  |
| **1.0-3.0** | 2,303 (37.0) | 793 (38.3) |  |
| **≥3.0** | 1,924 (30.9) | 568 (27.4) |  |
| **Chemotherapy** |  |  | 0.216 |
| **IMRT alone** | 828 (13.3) | 308 (14.9) |  |
| **CCRT** | 2,556 (41.1) | 814 (39.3) |  |
| **NACT+CCRT** | 2,679 (43.1) | 904 (43.6) |  |
| **CCRT+ACT** | 156 (2.5) | 47 (2.3) |  |

**^†^**Statistical comparisons between the training cohort and validation cohort were computed using the Chi-square test or Fisher’s exact test. A *P*-value of 0.05 indicates a significant difference. *According to the 8^th^ edition of the AJCC/UICC staging system; **Abbreviations**: WHO, World Health Organization; EBV DNA, circulating cell-free Epstein–Barr virus deoxyribonucleic acid; HGB, hemoglobin; LDH, lactate dehydrogenase; ALB, albumin; CRP, C-reactive protein.

**Supplementary Table 2:** Summary of multivariate Cox proportional hazard regression analysis of independent risk factors for early/late tumor progression.

| **Risk factors** | **Independent risk factors for**  **Early tumor progression** | | **Independent risk factors for**  **Late tumor progression** | |
| --- | --- | --- | --- | --- |
|  | **HR (95% CI)** | ***P value*** | **HR (95% CI)** | ***P value*** |
| **Age** |  | < 0.001 |  | < 0.001 |
| ***18–29 vs. < 18*** | 1.160 (0.457-2.941) | 0.755 | 5.364 (0.736-39.076) | 0.097 |
| ***30–39 vs. < 18*** | 1.860 (0.760-4.550) | 0.174 | 5.819 (0.811-41.755) | 0.080 |
| ***40–49 vs. < 18*** | 1.835 (0.753-4.474) | 0.182 | 7.673 (1.073-54.865) | 0.042 |
| ***50–59 vs. < 18*** | 1.623 (0.662-3.976) | 0.290 | 9.050 (1.264-64.777) | 0.028 |
| ***≥60 vs. < 18*** | 2.416 (0.983-5.942) | 0.055 | 12.825 (1.788-91.991) | 0.011 |
| **Gender *(Female vs. Male )*** | 0.735 (0.618-0.873) | < 0.001 | / | / |
| **Cigarette consumption** | 1.259 (1.089-1.455) | 0.002 | / | / |
| **WHO histologic type** | 0.509 (0.364-0.712) | < 0.001 | / | / |
| **T stage** |  | < 0.001 |  | < 0.001 |
| ***(T2 vs. T1)*** | 1.686 (1.229-2.312) | 0.001 | 1.203 (0.883-1.639) | 0.242 |
| ***(T3 vs. T1)*** | 1.656 (1.250-2.194) | < 0.001 | 1.368 (1.054-1.775) | 0.018 |
| ***(T4 vs. T1)*** | 2.155 (1.604-2.895) | < 0.001 | 1.982 (1.501-2.616) | < 0.001 |
| **N stage** |  | < 0.001 |  | 0.003 |
| ***(N1 vs. N0)*** | 1.182 (0.892-1.567) | 0.244 | 1.217 (0.925-1.601) | 0.160 |
| ***(N2 vs. N0)*** | 1.500 (1.112-2.022) | 0.008 | 1.556 (1.157-2.091) | 0.003 |
| ***(N3 vs. N0)*** | 1.921 (1.410-2.617) | < 0.001 | 1.551 (1.121-2.147) | 0.008 |
| **EBV DNA load (*< 2000 vs. ≥ 2000* copy/mL)** | 2.762 (2.307-3.306) | < 0.001 | 2.707 (2.263-3.238) | < 0.001 |
| **CRP** |  | 0.001 | / | / |
| ***1.0-3.0* vs. *<1.0* mg/L** | 0.982 (0.811-1.189) | 0.852 | / | / |
| **≥3.0 vs. *<1.0* mg/L** | 1.321 (1.096-1.591) | 0.003 | / | / |
| **LDH *( ≥245 vs. <245 U/L)*** | 1.347 (1.090-1.663) | 0.006 | 1.488 (1.192-1.858) | < 0.001 |
| **ALB *( ≥40 vs.<40 g/L)*** | 0.741 (0.606-0.907) | 0.004 | 0.634 (0.513-0.783) | < 0.001 |

**Abbreviations**: HR, Hazard ratio; CI, confidence intervals; EBV DNA, circulating cell-free Epstein–Barr virus deoxyribonucleic acid; CRP, C-reactive protein; LDH, lactate dehydrogenase; ALB, albumin.

***Supplementary* Figure 1**. **Forest plots showing association between clinicopathological characteristics and early tumor progression in nasopharyngeal carcinoma obtained using the univariate analysis. Abbreviations:** WHO, World Health Organization; EBV DNA, Epstein–Barr virus deoxyribonucleic acid; HGB, hemoglobin; LDH, lactate dehydrogenase; ALB, albumin; CRP, C-reactive protein.


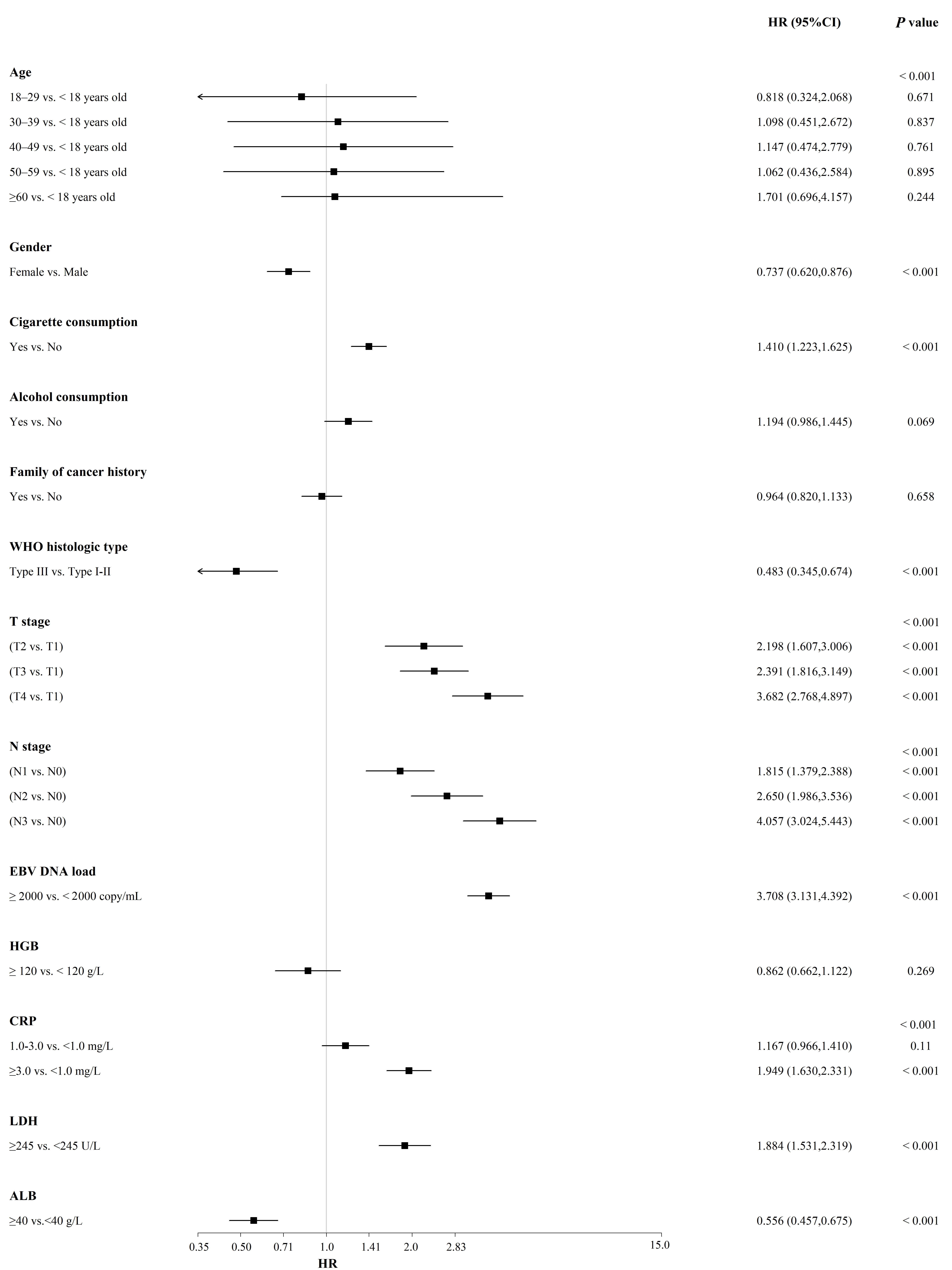


***Supplementary* Figure 2**. **Forest plots showing association between clinicopathological characteristics and late tumor progression in nasopharyngeal carcinoma obtained using the univariate analysis. Abbreviations:** WHO, World Health Organization; EBV DNA, Epstein–Barr virus deoxyribonucleic acid; HGB, hemoglobin; LDH, lactate dehydrogenase; ALB, albumin; CRP, C-reactive protein .


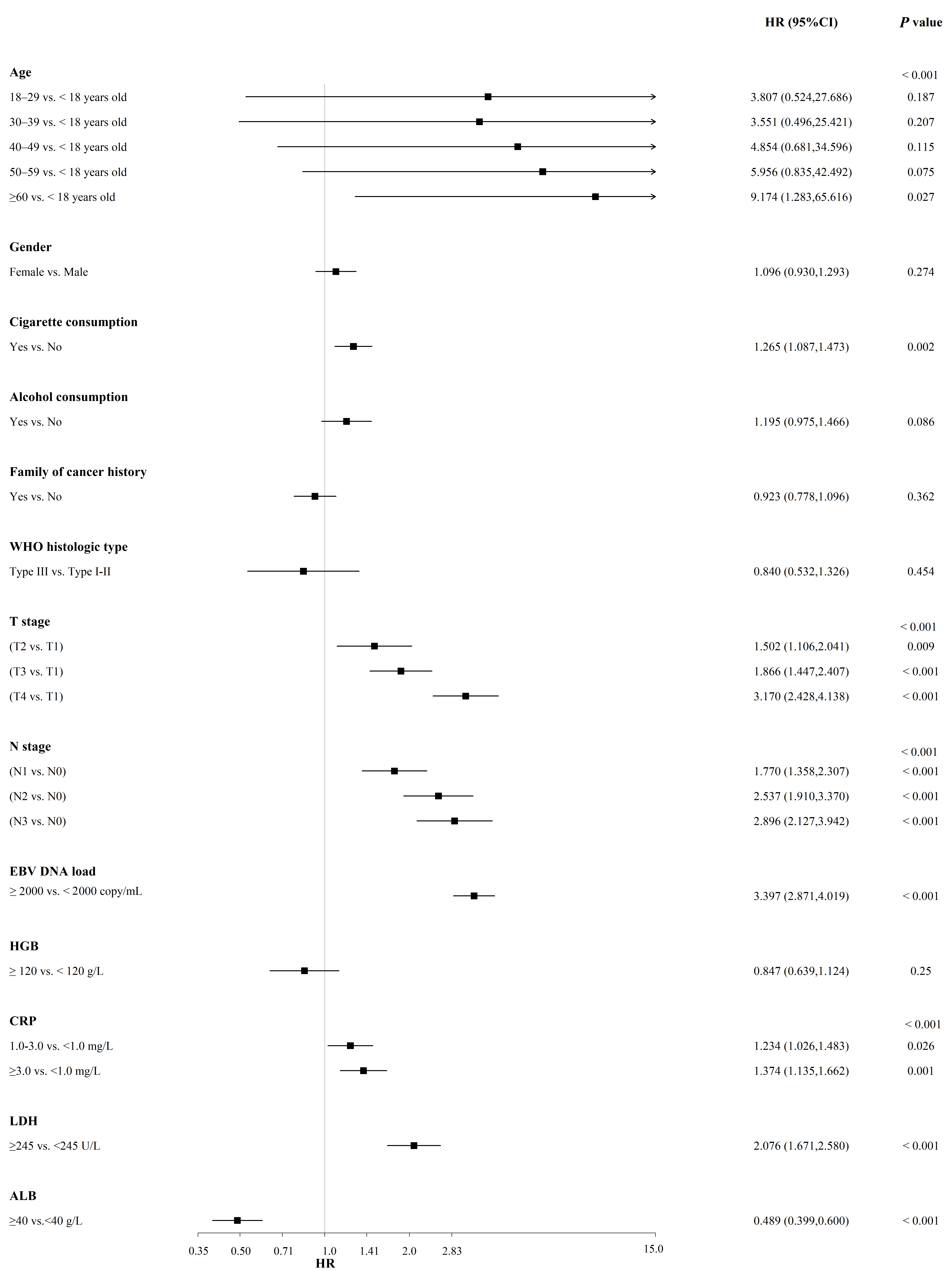


***Supplementary* Figure 3. Optimum cut-off values for the ETP-nomogram defined scores calculated using X-tile software**

The optimum cut-off value for the ETP-nomogram defined score was determined using an X-tile plot in the training cohort. The strength of the association between the score and PFS was visualized using different colors on the plot. The red color represents an inverse association between PFS and the ETP-nomogram defined score, and green color indicates a direct association. **Abbreviations:** ETP, early tumor progression; PFS, progression-free survival.


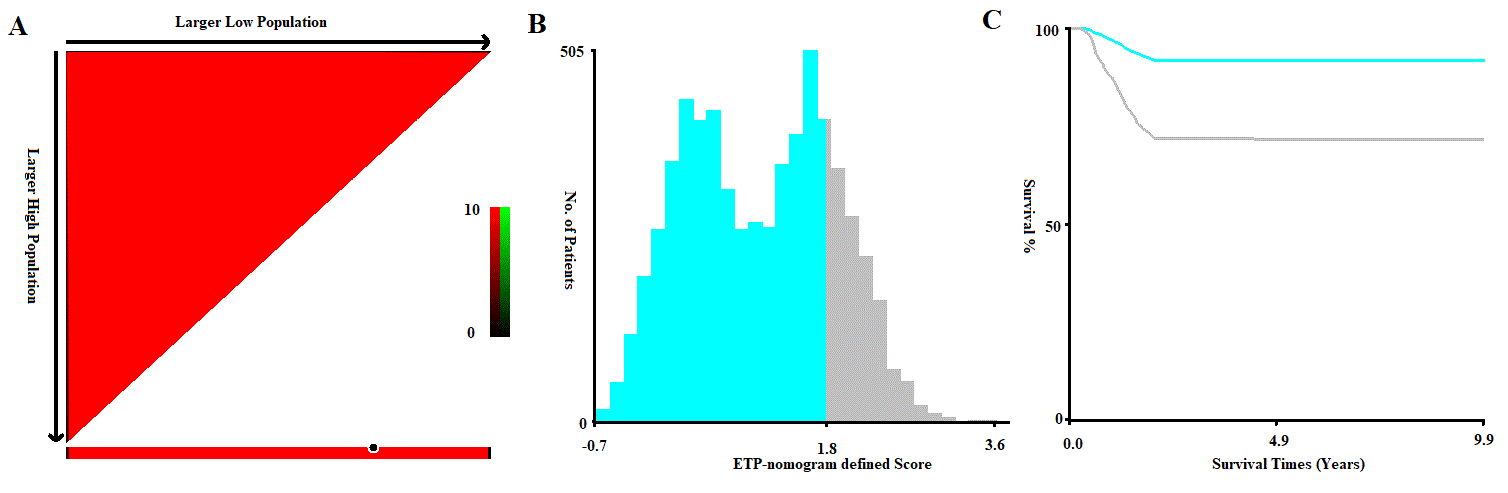


***Supplementary* Figure 4. Optimum cut-off values for the LTP-nomogram defined scores calculated using X-tile software**

The optimum cut-off value for the LTP-nomogram defined score was determined using an X-tile plot in the training cohort. The strength of the association between the score and PFS was visualized using different colors on the plot. The red color represents an inverse association between PFS, and the ETP-nomogram defined score, and green color indicates a direct association. **Abbreviations:** LTP, late tumor progression; PFS, progression-free survival.


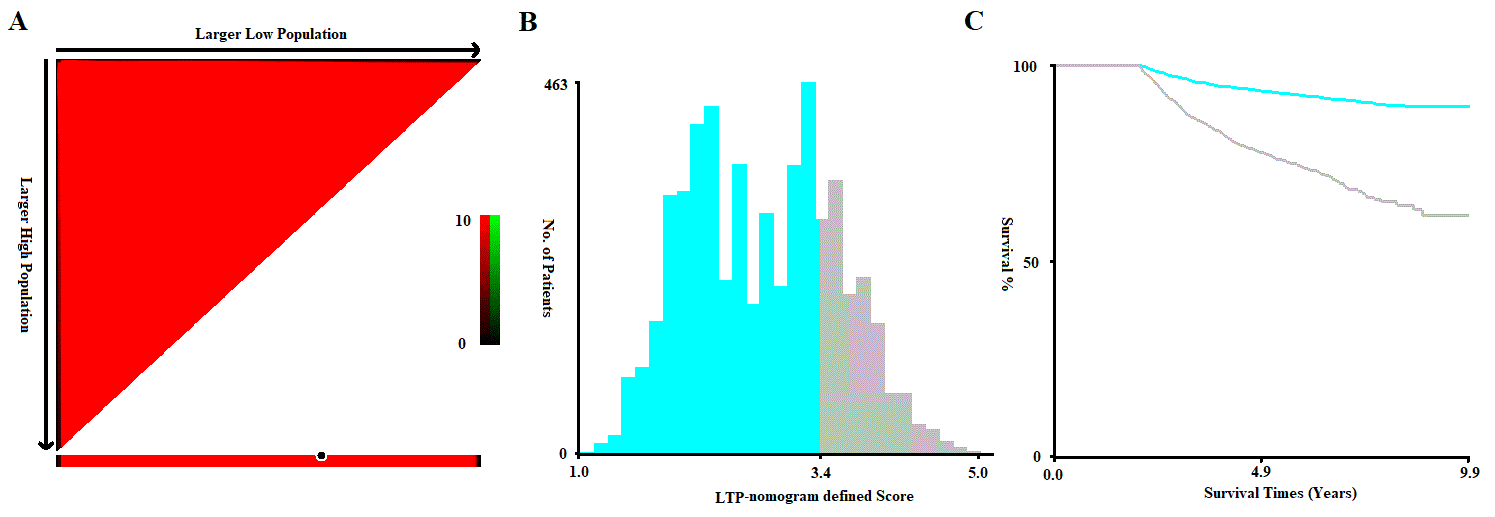

Supplement: Supplementary file 1 — Data S1 [file CAM4-12-5384-s001.docx]
